# The erratic nature of goodness: a reading of the parasitic nature of toxic masculinity through the character of Achuthan Nair in the select film duology

**DOI:** 10.3389/fsoc.2026.1815556

**Published:** 2026-05-18

**Authors:** Ajay Thomas Joy, R. Preetha

**Affiliations:** Department of English, School of Social Science and Language, VIT Chennai, Chennai, India

**Keywords:** identity, Malayalam films, masculinity, performance, society, traditional gender roles

## Abstract

The practice of masculinity can often masquerade as “goodness” in society. However, the underlying parasitic nature of this ideological practice can extend its sway over society, destroying the environment and the host it inhabits. This article examines the nature of masculinity, which preys on both the environment and its host in the Malayalam films *Kireedam* (1989) and *Chenkol* (1993), written by AK Lohithadas, directed by Siby Malayil. This perspective is drawn through the character arc of Achuthan Nair, played by Thilakan; his transition from a proud man to a loathsome end reflects the nature of toxic masculinity. This article on Connell’s hegemonic masculinity and Gramscian ideas of hegemony, coupled with an outspoken feminist performance by the female characters, critiques masculine ideals. This thereby repositions the tragic hero status from Sethumadhavan to Achuthan Nair. He acts as the fulcrum of the entire events of the films; “goodness” practiced by him has a performative quality approved by society. The statements, interactions, and discourses of Achuthan Nair act as the catalyst and medium of this destructive turn. The traditional gender roles and performances are also visited to identify the liminal nature of the host’s identity, which remains in flux, leaving him in an ideological limbo. The article tries to analyze the interactions of Achuthan Nair with other characters and situations to bring in this perspective of the parasitic nature of masculinity, with an emphasis on Achuthan Nair’s interactions with different forms of masculinities in the film, to represent the underplayed notion of hegemonic masculinity and its parasitic nature in a cult film duology. The transition of Achuthan Nair from a hegemonic practitioner to a victim of the same practice is also traced in the article.

## 1 Introduction

Malayalam cinema has maintained an eclectic approach to narrative throughout the years, with experimental and unconventional stories in the commercial, parallel, and art film spaces. As a star-driven industry, it holds its own idealized images and narratives that keep it together. The engagement with stereotypical characters and gender roles is one of those ideals. In the 1980s and 1990s, Malayalam cinema was predominantly engaged with the life of the commoner. The joys, troubles, and pain of the commoner got represented. Within this representation, the utmost space was occupied by the middle-class Malayali. Mainstream directors like Priyadarshan, Siby Malayil, and Sathyan Anthikad were mostly concerned about the life and struggles of the middle-class man. The most repeated face of this commoner was that of the veteran actor Mohanlal. Apart from the playful touch of the other two directors, director Siby Malayil’s narratives were always shaded by the spaces of pain, anguish, and trauma. With his close association with the writer AK Lohithadas, the stories take frantic turns, ranging from the author’s father’s desire to a tale of intergenerational madness passed down, and the narratives are so intense and heart-wrenching. The central characters in Lohithadas’s world are wounded. The characters are wounded from the start of the narrative, but his elusive, layered screenplays build in the audience the impression that everything is fine at the start. The pain carried by them is like a terminal illness that eats them up at the end. Sethumadhavan in *Kireedam* (1989) and *Chenkol* (1993), and Balan in *Thaniyavarthanam* (1987) are examples. His characters are weighed down by grief and sorrow, the reasons being the cultural and societal order, it demands and stereotyped ideals. The deaths in his films are tragic culminations of the socially approved toxic ideals, which are always transfigured as goodness.

Specifically, these narratives are loaded with ideological apparatuses. The film duology *Kireedam* (1989) and *Chenkol* (1993), written by AK Lohithadas and directed by Siby Malayil, is often read as a tragic tale of Sethumadhavan played by Mohanlal. His life and the unfortunate turn it takes. However, on a broader read, this offers a space to understand the practice of hegemonic masculine ideals in a familial space and how they are narrated and navigated within a familial structure often colored by the image of goodness. Connell’s idea of hegemonic masculinity reframes the film’s narrative. It repositions the character of Achuthan Nair, played by veteran actor Thilakan, as the sole fulcrum on which the entire story is narrated.

Throughout the film, the practices of Achuthan Nair are being legitimized by the system, which approves them as parental care and duty. The idea of goodness also plays in here. In Kantian ethics, goodness is paralleled with goodwill; he says that the quality of goodness is characterized by the quality of goodwill associated with it (Kant, 1998). Throughout the definitions and perspectives, the common good and the avoidance of violating another person’s life remain the common ground. However, here we see this as a playground of violations and violence. Achuthan Nair’s exercise of power, as narrated by the hegemonic system, unfolds in three phases: primarily with his son; second, with female characters, with particular importance of his daughter, Latha; and finally, his engagements with other forms of masculinities. It is interesting to note the double life of women characters in film; it is similar to that of women in a patriarchal structure, where they are women playing the wagon or true apostles of this ideology. They practice these and also transmit them to the next generation. Bell hooks talks about this in her idea of ‘Internalized Sexism’ as, “Internalized sexism leads women to police other women’s behavior in order to secure male approval, thus making them agents of patriarchal domination” (Hooks, 2004, p.38).

The selection of the select films is tied to the cult status in both commercial and artistic circles, and to their underplayed politics. The duology is a classic example of a well-crafted tragedy to this day, in which the actual tragedy is misrepresented. However, in the artistic and academic discourse, the performance of Sethumadhavan is kept at the center, and the downfall is tied to his actions; the nature of hegemonic masculine performances by the character of Achuthan Nair is the core of this deceptive, tragic outburst in the film. By formulating an idea of the parasitic nature of masculinity, Achuthan Nair’s image is brought to the center. Given its parasitic nature, the study examines the particular nature of hegemonic masculinity, which plays a double life in a person; here Achuthan Nair is the primary victim and primary practitioner of the hegemonic masculinity, which holds a toxic demeanor, blanketed in the image of goodness. The study seeks to unravel this practice of masculinity in the character and his interactions with other characters. This performance is being categorized as the character’s goodness or as the character’s responsibility within a social circle, but the study seeks to prove its hegemonic, toxic, and destructive nature and presents it as a product of a broken system. These types of camouflaging or disfiguring of toxic nature are even prevalent in current cinema.

The article tries to represent the practice of hegemonic masculinity by Achuthan Nair, thereby flipping the card of a second-fiddle character into primary space. The fluid or erratic nature of goodness through the close reading of the canonical film duology in Malayalam cinema*. Kireedam* (1989) and *Chenkol* (1993), directed by Siby Malayil and written by AK Lohithadas, are being done. The article situates Achuthan Nair as the victim and violator of the same ideology, giving the parasitic nature of hegemonic masculinity in a shared space. It also questions traditional gender roles from Achuthan Nair’s perspective. The female discourse of power is a male-dominated narrative, and their struggle for agency is also represented. Finally, the phallocentric concepts of goodness and common sense are also redefined in the discussion.

Research on masculinity and Malayalam cinema is gaining momentum, but select films remain underexplored in this area. Thomas and Manalil (2025) have employed *Kireedam* (1989) as one of the select films alongside *Taare Zameen Par* (2007), *The Pianist* (2002), and *Schindler’s List* (1993) in their work, *Framing emotions: using cinema as a tool for emotional intelligence in education*. B Shibu has also compared *Kireedam* (1989) to Narasimham (2000) in *Actor’s Age and the Production of Multiple Masculinities in the Context of Malayalam Cinema*.

Being the visible device of plot progression and center of the film, the narrative always keeps its edge for the “Righteous” constable “Achuthan Nair,” played by Thilakan. Achuthan Nair is the fulcrum on which the whole narrative of both films is marked. The tragic closure of all the characters in both films is silently navigated through Achuthan Nair’s character arc. He plays the initial reason for all the violent strife in the film, apart from his physical presence as the sole catalyst of that event, and the remaining characters and audiences are made to sympathize with him throughout the complete arc of his life till death. This article examines the hegemonic control that runs deep within the narrative, uncovering the systemic practices of control and how the sympathetic nature of the main characters transforms into a social evil, creating collateral damage. The study employs textual analysis of both films to incorporate a reading grounded in the discussion of masculinity. The discussion of hegemonic and toxic masculinity is placed at the center of the analysis of the character of Achuthan Nair.

## 2 Literature review

Connell and Messerschmidt (2005), in *Hegemonic Masculinity: Rethinking the Concept*, discuss the plurality of masculinity and how certain specific masculinities are respected and approved despite being destructive and repressive. Looking through this lens will provide a deeper understanding of the character of Achuthan Nair in the film duology compared with other masculine characters.

Gramsci (1971) in the book, *Selections from the Prison Notebooks*, discusses the concept of hegemonic control and the generation of consent through culture. This provides a deeper outlook on the constructed nature of consent and its normalized existence. The Gramscian notion of hegemony, coupled with masculine practices, could provide a deeper insight into the character of Achuthan Nair as the center of the tragic dip in the narrative.

A recent study in the field of masculinity by Sculos (2017) “Who’s Afraid of ‘Toxic Masculinity’?” gives a deeper understanding of the concept of “toxic masculinity” and offers a more contemporary outlook. He applies an autoethnographic approach in the study and places a conversation between extreme opinions. It shows the performance of masculinity as a regressive practice embedded with misogyny and violence. All opens more conversations on the topic of multiple masculinities. The definitions and concepts progression addressed by Sculos align with understanding the nature of such a masculine performance as parasitic.

In the conversation on the engagement of masculinity with the female characters and their responses to their practices, Pheterson (1996) narrates this in the book, *The Prostitute Prism*. The book’s primary focus is to offer a feminist critique of the practice of masculine ideals. This provides an understanding of the engagement with the idea of prostitution and how it is being weaponized against women; she also brings in a varied take on this concept. The construction of the term prostitute and narratives about prostitution are being categorized as tools of power that act as a tool for subordinating women in society. The stigma around this term is also being discussed in the book.

Bourdieu (2001), in the book, *Masculine Domination*, discusses the nature of masculine domination; he emphasizes the use of natural and good qualities associated with this controlled practice. In a discussion on the topic of masculine performances in the demography of Kerala, Devika (2007). In her book, *En-gendering Individuals: The Language of Re-forming in Twentieth-century Keralam*, she brings in the notion of gendered subjects, an identity which is beyond biology. She draws on the tools that apply this practice of gendering in society, such as norms, institutions, and media. She also talks about the role of language in the process of gendering.

Hooks (2004), in the book, *The Will to Change: Men, Masculinity, and Love*, contributes to the discussion of the notion of men and their gender roles. She puts forth the societal demands and requirements stemming from a social understanding of men as more functional than emotional.

## 3 Methodology

The study follows a qualitative approach that focuses on close textual analysis. The Malayalam film duology *Kireedam* (1989) and *Chenkol* (1993) is being studied within the theoretical framework of hegemonic masculinity proposed by Connell, with an emphasis on hegemonic and toxic masculine performances. The films are closely read, with a comparative understanding to trace Achuthan Nair’s progression through the different arcs of his life, thereby mapping a more detailed understanding that unravels the deepest roots of hegemonic masculinity, which is transfigured into the image of goodness. The negotiation between different masculinities and interaction with female figures in the films demonstrates the transitions and details of the toxic performance of the hegemonic masculinity.

This study discusses the cultural and systemic violence propagated by a hegemonic ideological structure. These lenses provide an organic interpretation of this systemic violation that employs a dual life to the ideological shackle. The primary conversation is about Achuthan Nair and his discourse in the film. Gramsci’s idea of hegemony is used to support and define the hegemonic masculine practices. Female characters and their voices are also represented through conversations about gender structures and roles in society. The employment of textual analysis offers a wide scope for understanding the films. It treats the selected films as readable texts that hold meaning as structured texts. This approach also supports the decoding of the embedded themes and codes that recur throughout the films. Character arcs, ideological undertones, power relations, and structural and thematic patterning in films through textual analysis, a close reading. It also provides scope for applying and integrating the theory of masculinity to textual evidence to yield concrete interpretations. This structured textual approach adds to the trustworthiness of the data presented.

The article examines the idealized form of violence in a shared domestic space, where it masquerades as goodness. Films are being placed as ideological wagons that hold the power to narrate a hegemonic system. The qualitative analysis of the films is narrated through the structure of the plot, narrative divisions, dialog, and mise-en-scène. These parts of the films act as terrains for the discussion of systemic violence and the gender and gendered violences. The gestures, performances, and dialogs with subtle silences give a micro-level understanding of the characters’ suppression and submission. The recurring themes of violence, violation, suppression, subordination, interpersonal conflict, vulnerability, and honor add deeper insight into the character of Achuthan Nair.

## 4 Analysis and discussion

### 4.1 Three phases of tragic violation

The article presents a tripartite study; the initial discussion is about Achuthan Nair’s hegemonic masculine practice in shaping his son, Sethumadhavan’s, life and choices. Second, the discussion is narrated around the space of subordinate masculinities and their engagement with Achuthan Nair’s power exchange, and third, the discussion focuses on the women characters, with emphasis on the character of Achuthan Nair’s daughter, Latha. In all these scenarios, violence barely comes as a tool for the application of this hegemonic control, according to Connell and Messerschmidt: “Hegemony did not mean violence, although it could be supported by force; it meant ascendancy achieved through culture, institutions, and persuasion” (Connell and Messerschmidt, 2005, p. 832). As mentioned, rather than being acquired by a male, it is ascribed to a man by the systems, social orders, and institutions that profit from his actions. The practice of hegemonic masculinity holds systemic power, which insidiously approaches and envelops its subjects. It’s important to note that the impact and negative residues left by this toxic ideology are not limited to women alone; men are also not immune to its toxic touch.

#### 4.1.1 Sethumadhavan: the tragic casualty

From a mellow, lovable boy next door to a wounded, kind-hearted criminal, who commits crimes to survive. When Sethumadhavan looks through the caged window of the upper room, his gaze falls to the close-lying street. He tells his friend, “That is the street, where Sethumadhavan lost everything, all his dream and even life” (Malayil, 1989, 01:32:11–01:32:26). The audience also believes in that reflection, but it was just the final blow that broke the Stone. Sethumadhavan is a subordinate subject to the society and its demands and conventions. Primarily, he is under his father’s control, Achuthan Nair. The authorial power of Achuthan Nair narrates all the actions, dialogs, and even guilt. Sethu broke out on that street onto those goons (gang members of Keerikadan Jose), in a psychoanalytic space; it was him trying to break out of the societally constructed image of Sethu, the ideal man with compliance. He uses the incident of his father getting assaulted to break the shackles on him. Here is a clear example of a father’s hegemonic control over his son. In the context of Kerala, masculinity holds a similar ground to honor.

The saddest part is that Sethu is consciously unaware of this reality, and until his tragic end, he valorizes the forceful control of his father figure. He lived and died as a subordinate subject. Here, Achuthan Nair’s interactions, in which he raises concerns about Sethu, linking them to his siblings’ lives and his father, highlight the social damage Sethu inflicts on his family; here, the character of Achuthan Nair is immune to holding responsibility for these damages. Sethu is a gentle and kind-hearted soul; it is evident that he is not a good match for a police job, but we do not even see a glimpse of any other possibility of a job being mentioned throughout the film- he was conditioned to a point to believe that being a sub-inspector (SI) would be the fine destination of his success.

Sethu is a disabled character in various ways; his ability to think and navigate right and wrong has been castrated by his father figure. Nothing comes from him other than what his father placed in him. Hooks in the book, *The Will to Change: Men, Masculinity, and Love*, says that “Patriarchy demands of men that they become and remain emotionally disabled. Since it is a system that denies men access to their full emotional being, it is a system that kills men” (Hooks, 2004, p. 17). Here, Sethu becomes a vessel that carries the father’s ideological fetus, which takes shape and form through Sethu. In many places, Sethu aligns with the image of the female psyche in the film, who are initially represented as carriers of both life and hegemony. According to Gramsci, “Hegemony is characterized by the combination of force and consent, which balance each other reciprocally, without force predominating excessively over consent” (Gramsci, 1971, p. 80). Here Achuthan Nair offers the consent of the characters, social structures, and institutions, which convince them to keep the order in his hand; in the case of force, it is his profession that holds that privilege. Being an instrument and part of the police force demands unearned respect, and compliance falls into the character of others.

The nature of goodness could be brought into question here; nobody in the film questions Sethu or his compliance, and all are happy with his state of subordination. Sethu was never born, although until his mid-twenties, he was carried inside the womb of his father’s ideals and dreams. When he fights the goons in the street, he forcefully breaks the ideological structure of his father’s control- because it is the last thing Achuthan Nair (father) wanted of his son.

The audience views the stunt in the street as the primary step in Sethu’s downfall, but from the opening of the film, he is a dying figure who finds it hard to breathe in his father’s air. Preparing for the SI test, he promises his father that he will watch his diet, but fails to meet the standards. When Achuthan Nair learns of it, he sulks and feels embarrassed. Achuthan Nair’s response is shocking: “Don’t give promises that can’t keep” (Malayil, 1989, 00:16:00–00:16:05). He says this with a slight smile. Sethu falls into guilt, a man in his 20s, feeling down for such a minor action, talks about the authorial control of the father figure. His pathetic part is that his father even narrates his guilt. Apart from the film’s bloody actions, these ideological cruelties are even more violent. The tool of shaming applied by Achuthan Nair is so powerful that it differently attacks Sethu’s psyche and moral conscience. Hooks (2004) discusses this idea of control, referring to it as control maintained through the tools of psychological terrorism.

Sethu, who dreamt of a fine life as a Police officer with Devi (his love), is no more. He is a known henchman of the town. Kafka’s traveling salesman Gregor Samsa found himself as a bug on a fine morning, which is the same that happened to Sethu. A single event in his life that brought a complete change. However, it holds a cumulative nature to that eruption. The birth/metamorphosis of the two creatures was instant, but they were carried, nurtured, and pregnant for years. The first half of the film *Kireedam,* (1989) is seen as a joyful ride, but the first scenes themselves mark the beginning of the tragedy. There is tragedy throughout the film; in the first half, it masquerades as “goodness.” Achuthan Nair is angry, and he channels his anger as a tool for the application of his power. Still, it distance him from others in many ways, bell hooks talks about anger “Patriarchy is a political-social system that insists that males are inherently dominating, superior to everything and everyone deemed weak, especially females, and endowed with the right to dominate and rule over the weak and to maintain that dominance through various forms of psychological terrorism and violence” (Hooks, 2004, p.58).

The Shift from Sethumadhavan to Ramapuram Sethu is the result of a struggle between traditional goodness and truth. It is very heart-wrenching that Sethu lost everything he had as a social animal: his family, his love, and a fair future. However, what he gained was agency, the ability to make decisions about his life. The change is hard and painful, but that is the price that was paid for freedom. When Sethu took his life in his hand, the first thing he lost was the cushioning and comfort that the structures of subordination offered.

However, the tragedy does not stop there; his desire to return to a somewhat similar life is hauntingly following him. The past is the specter that haunts, but in Sethu’s case, his compliance nurtured through his childhood acts as the specter that strangles him to death and also forces him to reject agency. Derrida (1994) talks about this as a Specter can be absent and present at the same time. The transformation puts him into a more nuanced hegemonic structure. His longing for a new start and a fair life with his new love, Indu, also foregrounds the remaining stains of his subordination, but is colored by his agency. The goodness of the character marked Sethumadhavan, but his fatal flaw was that he was blind to the control and hegemonic web that his father had over him.

#### 4.1.2 Achuthan Nair: the social nemesis

A patriarch and a victim of his own pseudo-masculine self-respect. The opening scene of the *Kireedam* (1989) is a metaphor of the tragedy it offers. The sugar-coated narration has moved it to a place where both Sethu and the audience are at the mercy of this man, Achuthan Nair. The scene is a dream: Sethu, a sub-inspector, walks into the station where Achuthan Nair works and salutes his son. This is the dream and desire of this man, where all the other characters in the films are mere tools to make this happen. This itself is metaphoric of the character’s nature and the nature of the narrative. It is all about Achuthan Nair and his dream. When he mentions this event as “His Dream,” that is always an exclusive claim.

We see a world designed and narrated by Achuthan Nair where all others are mere subjects to his order. When he says, “I never have hurt my children” (Malayil, 1989, 00:11:07–00:11:11), and yet everything falls into place as he wishes, this is a clear example of how hegemony works. Neo-Gramscian theorists describe hegemony as the capacity of leading groups to present their particular interests as the universal interests of society, thereby winning consent and exercising material power (Cox, 1983). Here, Achuthan Nair and his dream are presented as the common, selective good of individuals and families. He is the hegemonic force that functions as the root cause and practitioner of subordination in his social and personal space. The whole anarchist practices are masquerading under the face of unconditional love, selflessly sacrificing father, and epitome of truth and goodness.

Discussing “goodness” and its relative and erratic nature is important here. Achuthan Nair, being a law-abiding, honest officer, is being transferred as a punishment to Ramapuram, an infamous land of criminals. He is honest in his work, which does not give him the right to lay a hand on another person’s body. In that event, and later, the fight that breaks out with Keerikadan is more than just a matter of his duty. He is a man under the patriarchal structures. Regardless of his lower rank, he exercises his power through physical practice. In a way, his condition is very similar to castration anxiety.

Like in Freud’s theory, castration anxiety is the young boy’s unconscious fear that the father will punish his Oedipal wishes by removing his genitals, a danger first imagined when he notices the apparent “castration” of the female body. It is further developed to the point where this anxiety can be reformed into social anxiety. According to Freud, “Castration anxiety develops into moral anxiety—social anxiety. The first, that is, the infantile form of anxiety, is the reaction to the threat of losing the object of love” (Freud, 1959).

Achuthan Nair is at this point when his power and position are being questioned. For him, his ego is valued over his life. When he sees the officers who feel desensitized to the actions of criminals, Achuthan Nair is in fear; he forcefully acts to re-establish the power structure. Here, the object of love is the power structure from which he derives pleasure, comfort, and validation. Apart from loathing for this character, there is room for sympathy. He is also a victim of structural violence and hegemony. Going back to the goodness, the nature of goodness is being questioned here, from an officer who was not ready to comply with a man for parking in the wrong location, to a father who accompanies his own daughter for prostitution, is a tragic fall in morals and character. What brought him to that condition? In retrospect, it’s evident that it was the sole hegemonic pattern in his life that obliterated his and everybody’s lives around him. Both these films are also voices of structural violence. When Sethu learns about the off-putting condition of his sister, what breaks him is the revelation that all these happened under his father’s knowledge. The only scene where Sethu storms at his father, that is the destruction of all the structures of power Achuthan Nair held, and we never see that character alive. The fulcrum of power practice was Sethu, when he bashes back at him, Achuthan Nair is dead. It broke him more than his daughter being a prostitute. The law raises questions about power and goodness: primarily, is goodness in the traditional sense, or in the unfettered practice of power?.

The end of Achuthan Nair is not shown on the screen; it is just mentioned. “If an individual is not successful in emotionally crippling himself, he can count on patriarchal men to enact rituals of power that will assault his self-esteem.” (Hooks, 2004, p.74). The man who practiced this power is a disabled person now. Achuthan Nair encounters Sethumadhavan at a point where Sethumadhavan knows that his sister works as a prostitute and his father accompanies her, which breaks him completely. Achuthan Nair cannot bear the assault on his self-respect. Achuthan Nair vanishes from that point. The letter Achuthan Nair left for Sethumadhavan is the extent to which the control stretches his hand. Throughout both films, no one points the finger at him as the reason for all the colossal damage; the only scene where Sethu questions him is when Achuthan Nair ends his life. It also has a resistive nature to the hegemonic structure on both its practitioners and subjects.

The notion of parasitic masculinity suggests a nature of hegemonic masculine performance. It shows the double life of a person in this masculine performance. Here, the practitioner of such an ideology is the primary victim of the hegemonic practice, still being the practitioner. In the select films, Achuthan Nair is the primary victim and primary practitioner of the hegemonic system. Another trait of this masculinity is its self-destructive nature, which blinds its practitioners to the fact that they are the primary subjects of violation. The tragic end of Achuthan Nair addresses this notion. It preys on its own host and practitioner. Here, the tragic hero is not Sethumadhavan but Achuthan Nair, who is more violated.

#### 4.1.3 From loathing women to autonomous being

In the discussion of the female characters in *Kireedam* (1989) and *Chenkol* (1993), it becomes evident that the majority of them remain structurally flat and do not undergo a significant developmental arc over the course of the narrative. The role of women in the films is to play the second fiddle. They are represented as subjects of patriarchal control, and as subjects who accept and propagate the dominant masculine narratives. Hooks talks about it as “the role women play in perpetuating and sustaining patriarchal culture so that we will recognize patriarchy as a system women and men support equally, even if men receive more rewards from that system. Dismantling and changing patriarchal culture is work that men and women must do together.” (Hooks, 2004, p.43). From Achuthan Nair’s wife to his elder daughter, and even the female characters on the antagonist side are mere subjects of hegemonic control. The writer and director are not letting their female characters grow out of these shackles. They try to keep them in a binary understanding of Madonna/promiscuous person. Either they are virtuous women or the other extreme. This understanding of women in binary spaces limits the possibility of female characters to grow. Also, this acts as a hegemonic structure that limits them from becoming an autonomous being. They straitjacket them into the stereotypical understanding of women.

Women are represented as the victims of the same hegemonic masculinity that Sethu undergoes. However, the violation occurs at two levels for women. Throughout both films, we see women mostly in domestic spaces, rarely outside the house. Their movements are restricted to the household. Once they move out, they are guided by men. In the film *Kireedam* (1989), in the initial scenes, we see Devi going to teach; she is accompanied by her father. Even in another scene, Sethu asks his brother to accompany Devi back home. They are completely guided and guarded by men. Compliance is the response they take to this action. They fail to structure the system of hegemonic order.

The women characters are mere subjects, where symbolic violence is being instrumentalized. As Bourdieu puts it, “Symbolic violence is a form of power which is exercised upon a social agent with his or her complicity” (Bourdieu, 2001, p. 34). The agent that performs this power is the repressive structure of hegemonic masculinity. That is the tool that puts them into compliance through unconscious approval guided by an invisible force. The violated social agent here is the female subject. The production and approval created through compliance places them in a subordinate position where they will fully live. The violation of the knowledge and thinking system is the primary reason.

Two main characters who stand out from this crowd are Latha, Achuthan Nair’s younger sister, and Indu (Keerikadan Jose’s stepdaughter, who later becomes Sethu’s love interest). Latha, a young girl, has grown much over the past 7 years. In the first film, *Kireedam* (1989), we see her as a person who does not hold any agency over her life, completely succumbing to her father’s orders. However, in the second film, *Chenkol* (1993), she is the most evolved character. In *Kireedam* (1989), in the film’s initial part, there is a scene where Latha says behind a window, with Sethu and Achuthan Nair on the opposite side (Malayil, 1989). That narrated her life then, under the visible control of her father. She was behind the bars of the social order and patriarchal system. We barely see her outside the home or the places around it. Her life was tied to the system, which was made to make men’s lives easier. She takes charge of the scene in the second film. Primarily, she gets outside of the house. Being a drama artist, she also holds a double life as a prostitute. She does it out of her will to support the family. When Latha asks Sethu, “Chetta do you need some money? I have it with me now” (Malayil, 1993, 01:11:14–01:11:24). From that point, we never see Latha overshadowed by any character; she is the giver, the major provider of the family, so she holds the power. When she asks Achuthan Nair to wait while she gets ready for her job, it suggests the power shift. From a mere spectator of the masculine patriarchal performance to a voice that narrates their existence. Rameshan, the youngest son of Achuthan Nair, studies at Latha’s expense—a reversal of the provider cycle.

Even though Latha has grown out of her shackles, the male conscience remains regressive. Sethu’s desire is only to marry off Latha, even at the cost of hiding the dubious past of her life, but Latha rejects that proposition. We see that her second life as a prostitute is what gives others a say on her life, as Pheterson (1996) says the social labels like promiscuous person and deviant acts as categories which help masculine figures to control the structural boundaries of their power discourse, which keeps the above-mentioned women in control, and through this ideology, they spread the idea of a clean, respected society.

The second female character who offers resistance to the social order is Indu—the stepdaughter of Keerikadan Jose. The questions of morality, justice, and human worth are given new dimensions by her actions and stance toward Sethumadhavan. From initially nursing wounded Sethu from her doorstep, providing him a space to heal, and finally offering her approval for his love, this provides a more derived form of morality. She is not a character who is completely revived. However, she serves as a stronghold that rejects and opposes hegemonic masculine performance. The scene where she refuses to disclose information about Sethu to Jose’s brothers is representative of her demeanor, which rejects such a social order. We rarely see her outside; throughout both films, the space feels like a metaphor for complete liberation. The presence of women in the house, opposed to their leaving that protective hegemonic layer created by masculine ideals.

In the feminist understanding of these films, it is evident that violence is a part of women characters’ lives. They are ripped of their identity by making them reliant on a male system. We hardly see a woman wounded in the films in comparison with central male characters, nor are they involved in a brawl, but they are subjects of violence. The hegemonic control employed in their lives is resulting in tragic and physical violent outcomes, which marks the presence of toxic masculinity in their lives. Also, in the discussion of Achuthan Nair’s masculine performance, his daughter Latha’s character’s evolution plays a pivotal role in his fall from the hegemonic masculine grace to a man who wanders in guilt and self-shame.

## 5 Conclusion

The films *Kireedam* (1989) and *Chenkol* (1993) offer an account of moral decline. Apart from the personal, tragic flaws of a character, systemic violence acts as a tool for the destruction of its own subject. The study shifts the focus from Sethumadhavan as the tragic hero of the film to his father, Achuthan Nair, and shows how the societally legitimized actions of that man carried an undercurrent of systemic violence at both the personal and social levels. It also unravels the parasitic nature of hegemonic masculine performances, which prey on their host while spreading destruction in the environment. The topic of conditioning done by the system, which derails its subject, is also presented.

The study of the select films delivers an understanding of hegemonic masculinity and its toxic performances in the cultural milieu of 1980s and 1990s films. From the perspective of film studies, the study seeks to understand different layers and narratives of masculinity and its engagements with the characters in the films. Moving onto the dialogs, the study incorporates the performances, staging of the scene, mise-en-scène, and character placements to narrate the complex spectrum of the application of hegemonic masculinity and its toxic performances covered in the cape of social convention and universal goodness.

The key sequences of the films are being closely analyzed to show notions of honor, control, and fractured manhood. The image of a fractured, violated man is presented in both the characters of Sethumadhavan and Achuthan Nair, Sethu being the long-standing victim who culminates in a tragic end through the application of his agency. His death itself is not personal but a product of a fractured system. The self-destructive end of Achuthan Nair is a voice for the gravity and depth of this toxic violation. The primary subject of the masculine hegemony was never Sethu, but was Achuthan Nair. Rather than an impressionistic understanding of the films, the study addresses the visual semiotics that garners the understanding of hegemonic masculinity.

Achuthan Nair is the Vanguard of hegemonic masculinity in this space. His performance of it makes it appear as a serene and legitimate form of action because of hegemonic control. The deployment of hegemonic practices of Achuthan Nair is leading to a result that parallels the performance of toxic masculinity. The parental control, care, and devotion to his duty are all colored by the nature of toxic masculinity tied to its outcome. Rather than the free play of violence and physical brutality, Achuthan Nair cuts through people’s hearts through his gestures and words. The street fight with the goons also addresses his potential for physical violence.

As Achuthan Nair’s arc progresses, we see the character’s downfall from two perspectives: personal and social. The gravity of the personal tragedy he endured underscores the violent nature of the system he practiced. The unfolding of Achuthan Nair’s character represents the unfolding of the understanding of virtue and power in an authoritative space. A man who held complete agency over his own and others’ lives is now in the same struggle. The narrative progresses as Achuthan Nair transforms from practitioner to victim; these were not two completely different phases, but they co-existed at different levels. The parental practice employed by Achuthan Nair is not an isolated practice but part of a broader power configuration in the system that privileges phallocentric control. The nature of his job is power-driven; that force is carried into his familial spaces. Here, power, hierarchy, and control define Achuthan Nair’s parental practices and other interactions—morality and compliance with this hegemonic order are held in parallel within the system. The notion of being on the side of morality through compliance derails Sethumadhavan at every level of his life.

The question of identity is another active conversation in the films. Achuthan Nair and Sethumadhavan hold spots or act as terrains where multiple identities coexist; the only constant in their lives is their identity as subjects of a violating system. His father defines Sethumadhavan’s life; the collapse of that dream directly addresses the collapse of a patriarchal system. This space denies any growth or deviations prescribed that are outside the yardstick of the system. The primary discussion of hegemonic masculinity centers on overt discussions of masculine performance. The analysis of *Kireedam* and *Chenkol* adds to the existing definition by addressing masculine hegemony, which is socially and culturally approved. The order and goodness that were traditionally held as the moral narrative for running a system are being dismantled to reveal hidden threads of hegemonic violence underneath. This study offers a more comprehensive understanding of the social and cultural engagements of masculine performers through the close reading of select films, thereby contributing to the spectrum of understanding of men in the social spaces of Kerala.

## References

[ref2] Bourdieu P. (2001). Masculine Domination. Stanford, California: Stanford University Press.

[ref3] Connell R. W. Messerschmidt J. W. (2005). Hegemonic masculinity: rethinking the concept. Gender Soc. 19, 829–859. doi: 10.1177/0891243205278639

[ref001] Cox R. W. (1983). Gramsci, hegemony and international relations: An essay in method Millennium. J. Int. Stud. 12, 162–175. doi: 10.1177/03058298830120020701

[ref4] Derrida J. (1994). Specters of Marx: The State of the Debt, the Work of Mourning and the New International. New York: Routledge.

[ref5] Devika J. (2007). En-Gendering Individuals: The Language of re-Forming in Twentieth-Century Keralam. Hyderabad, India: Orient Blackswan.

[ref6] Freud S. (1959). “Inhibitions, symptoms and anxiety,” in The Standard Edition of the Complete Psychological Works of Sigmund Freud, ed. Strachey J., vol. 20 (London, UK: Hogarth Press), 75–176. Original work published 1926

[ref7] Gramsci A. (1971). Selections from the Prison Notebooks. New York: International Publishers.

[ref8] Hooks B. (2004). The Will to Change: Men, Masculinity, and Love. New York: Atria Books.

[ref10] Kant I. (1998). Groundwork of the Metaphysics of Morals. Cambridge, UK: Cambridge University Press.

[ref13] Malayil S. (1989). *Kireedam* [Film]. Kerala, India: Kripa Films.

[ref14] Malayil S. (1993). *Chenkol* [Film]. Kerala, India: Kripa Films.

[ref19] Pheterson G. (1996). The Prostitute Prism. Amsterdam: Amsterdam University Press.

[ref23] Sculos B. W. (2017). Who’s afraid of ‘toxic masculinity’? Class, Race and Corporate Power. Available online at: https://www.jstor.org/stable/48645481 (Accessed December 6, 2025).

[ref25] Thomas S. Manalil P. (2025). Framing emotions: using cinema as a tool for emotional intelligence in education. Front. Psychol. 16:1650676. doi: 10.3389/fpsyg.2025.1650676, 41159169 PMC12556816

